# Influence of Recycled Tire Steel Fibers on the Mechanical Properties and Carbon Emissions of High-Performance Cement-Based Materials

**DOI:** 10.3390/ma18133008

**Published:** 2025-06-25

**Authors:** Liqiang Wu, Chenxiang Feng, Ji Qiu, Longlong Wang, Yuan Peng, Jintao Liu

**Affiliations:** 1 Zhejiang Institute of Hydraulics and Estuary (Zhejiang Institute of Marine Planning and Design), Hangzhou 310020, China; wulq@zjwater.gov.cn (L.W.); pengyuan2799@dingtalk.com (Y.P.); 2 College of Civil Engineering, Zhejiang University of Technology, Hangzhou 310014, China; 221123060123@zjut.edu.cn (C.F.); jtliu@zjut.edu.cn (J.L.); 3 Zhejiang Guangchuan Engineering Consulting Co., Ltd., Hangzhou 310020, China; qj1000025@163.com; 4 Hangzhou Commerce & Tourism Group Co., Ltd., Hangzhou 310003, China

**Keywords:** SIFCON, UHPC, RSF, mechanical properties, carbon emissions, LCA

## Abstract

To address the issues of high carbon emissions from concrete and high energy consumption in the manufacturing of traditional steel fibers, this study investigates the feasibility of replacing industrial steel fibers (ISF) with recycled tire steel fibers (RSF) in high-performance cement-based materials. The study examines the effects of fiber type and dosage on the mechanical properties within the systems of ultra-high-performance concrete (UHPC) and slurry-infiltrated fiber concrete (SIFCON) and analyzes the carbon emission levels using the Life Cycle Assessment (LCA) method. Research results indicate that the compressive and tensile strengths of SIFCON are significantly higher than those of UHPC. Under the same conditions, RSF has little difference in tensile performance when compared with ISF, suggesting a great substitution potential. Carbon emission analysis shows that although the total carbon emissions of the SIFCON system are relatively high, its performance improvement is remarkable. Both the carbon emission per tensile strength and carbon emission per compressive strength are lower than those of UHPC, demonstrating a high degree of environmental friendliness. Overall, this study shows that RSF can not only effectively enhance the performance of high-performance cement-based materials but also reduce carbon emissions, making it a reinforcing material with both excellent performance and sustainability.

## 1. Introduction

As one of the most widely used building materials globally, concrete production consumes vast amounts of natural resources and is accompanied by significant carbon emissions [1]. Improving the mechanical properties of concrete can effectively reduce material consumption, achieving carbon emission reduction to a certain extent. With the increasing demand for high-performance concrete, scholars have started to explore the addition of steel fibers as reinforcing materials to concrete to enhance its mechanical properties and durability [2]. However, the high energy consumption and carbon emissions during the production of ordinary steel fibers limit their large-scale application. In China, approximately 330 million used tires are generated annually, equivalent to a weight of over 10 million tons, and the annual amount of discarded tires continues to grow at a rate of 6~8% [3]. Each discarded tire contains about 1.3 kg of steel fibers, which can be used as structural reinforcement materials, presenting high recycling value and potential application prospects [4].

Existing studies have shown that although recycled tire steel fibers (RSFs) vary in geometric shapes, their irregular morphology actually enhances the mechanical anchorage between the fibers and the matrix [5]. Through optimizing the fiber content and mix proportion design, the key mechanical performance indicators of recycled steel fiber concrete, such as compressive strength, tensile strength, flexural toughness, and impact resistance, can approach or reach the levels of industrial steel fiber (ISF) concrete [6,7,8]. It is worth noting that during the mixing process of ordinary steel fiber concrete, an excessive amount of steel fibers may lead to issues such as fiber segregation, clustering, high air content in concrete, and difficulty in exceeding a steel fiber volume fraction of 2%. Slurry-infiltrated fiber concrete (SIFCON), on the other hand, is formed by injecting mortar with certain fluidity into a mold pre-placed with a steel fiber skeleton. The volume fraction of steel fibers in SIFCON can be as high as 25% [9]. SIFCON can significantly improve mechanical properties, its tensile strength can be comparable to the compressive strength of ordinary concrete, and its energy absorption capacity is three orders of magnitude higher than that of ordinary concrete. Since SIFCON usually employs high-strength cement-based materials with a low water/cement ratio and a high fiber content, its compressive and flexural strengths are significantly higher than those of conventional steel fiber concrete. RSF can significantly enhance the tensile, flexural properties, and toughness of SIFCON while having a limited impact on its compressive strength [10]. As the fiber content increases, the flexural strength increases significantly, and the compressive strength also exhibits a smaller increase. Moreover, a higher fiber content creates a complex spatial structure among the fibers, resulting in a significant mechanical interlocking effect and causing a ductile failure mode [11,12]. In summary, RSFs demonstrate a significant potential to enhance the mechanical properties of concrete, and their structural contribution can be further amplified through the infiltration casting technique. Nevertheless, current research on the carbon reduction potential of RSFs on concrete remains limited, indicating a need for a more comprehensive and systematic investigation.

In recent years, RSFs have gained increasing attention in sustainable concrete due to their dual benefits of carbon reduction and solid waste valorization. Wang et al. [13] conducted a Life Cycle Assessment (LCA) and reported that UHPC incorporating 2% RTSF exhibited a carbon dioxide emission of 929.64 kg CO_2_/m^3^, which was 23.91% lower than that of UHPC made with ISF. Moreover, the corresponding carbon emission intensity (expressed by volumetric carbon emission per compressive strength) was also reduced by 18.1%. Similarly, Biswas et al. [14] reported that the carbon emission factor of RTSF is approximately 0.8 kg CO_2_/kg, which is about 22.3% lower than that of industry steel fibers while maintaining a comparable mechanical performance. These findings affirm the potential of RSFs to serve as an eco-efficient alternative in producing cement-based materials, contributing to both carbon emission reduction and circular economy goals.

Therefore, in this study, the effects of dosage and type of steel fibers on the mechanical properties of cement-based materials are first analyzed through experiment analysis, and the feasibility of RSFs in practical applications is explored. Secondly, the Life Cycle Assessment (LCA) method is adopted to analyze the carbon emissions during the processes from raw material production and transportation to pouring. Considering the influence of different functional units, the strengthening effect of steel fibers and their impact on the carbon emissions of cement-based materials are evaluated.

## 2. Experiment Analysis

### 2.1. Raw Materials and Mixture Proportions

#### 2.1.1. Cementitious Materials

(1) Cement

In this experiment, PⅡ 52.5 Portland composite cement (Conch (Zhejiang) Holdings Co., Ltd., Hangzhou, China)was used. Its basic properties are shown in Table 1.

(2) Silica fume (SF)

The silica fume used was the off-white KLKEN 920U, produced by IKEN International Trading Co., Ltd. (Shanghai, China), whose chemical composition and physical properties are shown in Table 2.

(3) Fly ash (FA)

Class I fly ash (Hangzhou Xiaoshan Jinlong Fly Ash Co., Ltd., Hangzhou, China) with a specific surface area of 540 m^2^/kg was used. Its chemical composition is shown in Table 3.

#### 2.1.2. Water Reducing Agent (WRA)

In this experiment, the Melflux 4930 F superplasticizer (BASF Construction Chemicals Co., Ltd., Trostberg, Germany) was used. The basic properties are shown in Table 4.

#### 2.1.3. Fine Aggregate

**Figure 1:**
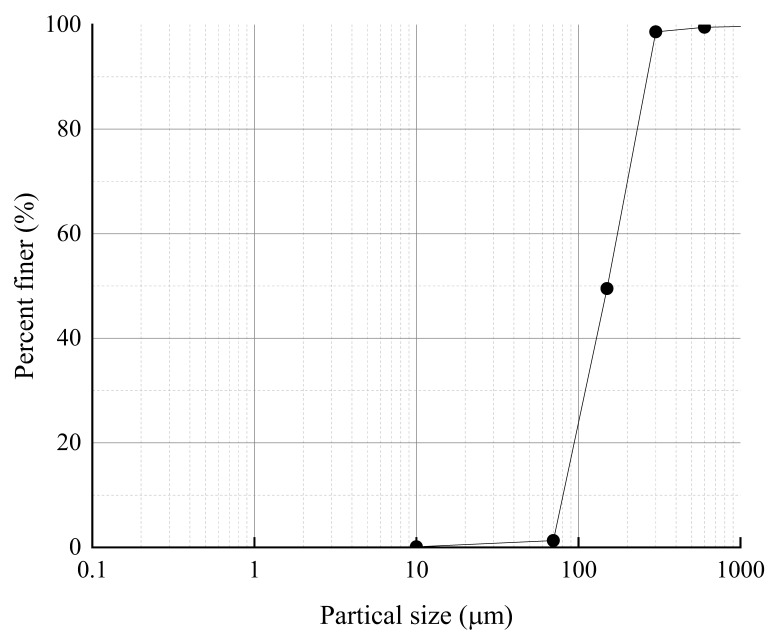
Grading curve of fine aggregates.

Sand (Shanghai Gangqi Building Materials Co., Ltd., Shanghai, China) with water absorption of 0.56%, moisture content of 0.43%, bulk density of 1450 kg/m^3^, and specific gravity of 2.63 was used as the fine aggregate. The grading curve is shown in Figure 1.

#### 2.1.4. Steel Fibers

**Figure 2:**
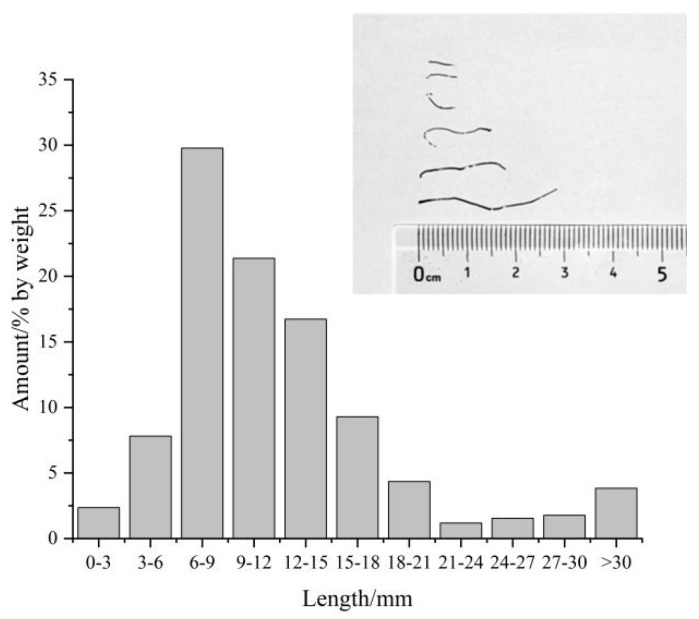
Shape and length distribution of RSFs.

The ISFs (Jiaxing Jingwei Steel Fiber Co., Ltd., Jiaxing, China) used in this study were straight in shape, without hooked ends. The waste tires are cut and crushed into small pieces, and the resulting product is screened to separate particles of different sizes. Subsequently, the recycled steel fibers are efficiently recovered using magnetic separation equipment [15]. Affected by the model of rubber tires, the lengths of RSFs vary a lot, and their shapes are non-straight. RSFs are produced by Shanghai Jinghan Co., Ltd., Shanghai, China. The shape and length distribution of RSFs are shown in Figure 2. The morphology and basic physical properties of RSF and ISF are shown in Table 5.

The mix proportion is shown in Table 6. To study the influence of fiber type and fiber dosage on the basic mechanical properties of cement-based materials, four groups of specimens are designed (Table 6). Among them, the S group is the SIFCON group, focusing on investigating the influence of fiber dosage, and the U group is the UHPC group, focusing on investigating the influence of fiber type. It is worth noting that the volume fraction of steel fibers in SIFCON can be far higher than that in UHPC. Therefore, the volume fraction of steel fibers in the SIFCON group and the UHPC group are different.

### 2.2. Specimen Preparation

#### 2.2.1. Specimen Size

According to the *Test Methods for Physical and Mechanical Properties of Concrete* (GB/T 50081-2019) [16] and the dimensional design approaches proposed by domestic and international scholars [17], under different curing days, for each specification, 3 cube compressive specimens with dimensions of 100 mm × 100 mm × 100 mm (12 in total) for compressive strength testing, 3 cylindrical compressive specimens with dimensions of Ø50 mm × 100 mm (12 in total) for compressive strength testing and stress–strain analysis, and 3 dumbbell-shaped specimens (12 in total) for uniaxial tensile test are cast. That is to say, each group contained three replicates per test, which aligns with common practice in experimental concrete research. The dimensions of the gauge section in the middle of the tensile specimen are 90 mm × 40 mm × 50 mm. The specimen mold is made of No. 45 steel and mainly consists of one bottom plate and two side plates. The thickness of the bottom plate is 13 mm. The two side plates are connected by interlocking and fixed on the bottom plate with socket head cap screws to ensure the stability and accuracy of the specimen molding.

#### 2.2.2. Preparation Method

First, accurately weigh each component material according to the matrix mix proportion. Pour cement, fly ash, silica fume, and fine sand into the mixer in sequence for dry mixing for 5 min to ensure uniform distribution of each component. Subsequently, add the water reducing agent and water in the predetermined proportion and continue mixing for 8 min to make the paste uniform and in a state with good fluidity. For the SIFCON mixtures, dry steel fibers were pre-placed into the molds in a random or oriented manner and compacted to achieve the target fiber volume fraction prior to slurry casting. For the UHPC mixtures, water and WRA were added first, followed by the incorporation of RSFs and ISFs during the wet mixing stage. The mixture was stirred at medium speed for 3 min, then at high speed for an additional 2 min to ensure uniform dispersion of fibers. Finally, after pouring, let the specimen stand for 24 h and then demold it. Place it in a standard curing room at (20 ± 2) °C and a relative humidity of over 95% for curing. Take out the specimens after curing for 7 d or 28 d, respectively, and mark them well.

### 2.3. Test Methods

#### 2.3.1. Compressive Strength Test

**Figure 3:**
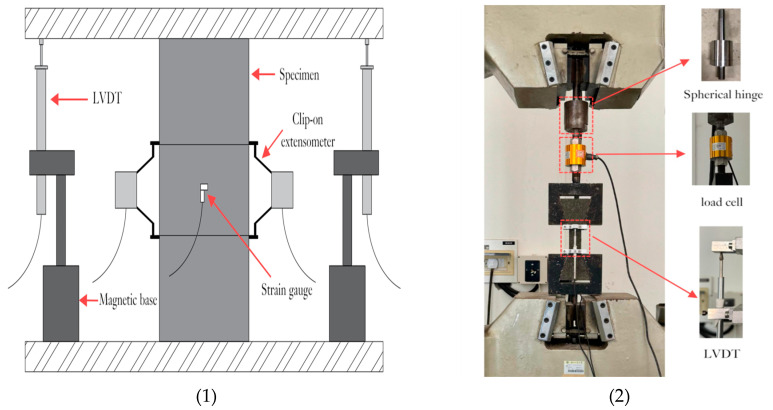
Loading and data collection: (1) cylindrical compression specimen, (2) tensile specimen.

Use the STYE-3000C compression testing machine (Zhejiang Geotechnical Instrument Manufacturing Co., Ltd., Shaoxing, China) to load the cube specimens. Through the EHC-1000 digital electro-hydraulic measurement and control system (Jiangsu Donghua Testing Technology Co., Ltd., Jingjiang, China), adopt force value control, and control the loading speed at 2 kN/s. Use the WAW-1000 microcomputer-controlled electro-hydraulic servo universal testing machine (Zhejiang Geotechnical Instrument Manufacturing Co., Ltd., Shaoxing, China) to load the cylindrical specimens. Adopt displacement control and control the loading speed at 0.2 mm/s. After grinding the front and rear surfaces of the specimens, stick strain gauges on them. Use a clip-on extensometer at the middle of the specimens to measure the displacement, which is 25 mm away from the upper and lower surfaces, respectively. Data is collected through the TDS-530 data acquisition instrument (Jiangsu Donghua Testing Technology Co., Ltd., Jingjiang, China). The schematic diagram of loading and data collection is shown in Figure 3 (1).

#### 2.3.2. Tensile Strength Test

The direct tensile method is selected to test the tensile properties. Adopt displacement control for loading and control the loading speed at 0.2 mm/s, with three specimens in each group. Use the TDS-530 data acquisition instrument to collect all the data. Connect a load sensor below the spherical hinge to measure the load. Fix the gauge section of the specimen with a fixture, and use a displacement meter to measure the displacement between the fixtures. The schematic diagram of loading and data collection is shown in Figure 3 (2).

## 3. Results and Discussion

### 3.1. Test Results of Compressive Performance

**Figure 4:**
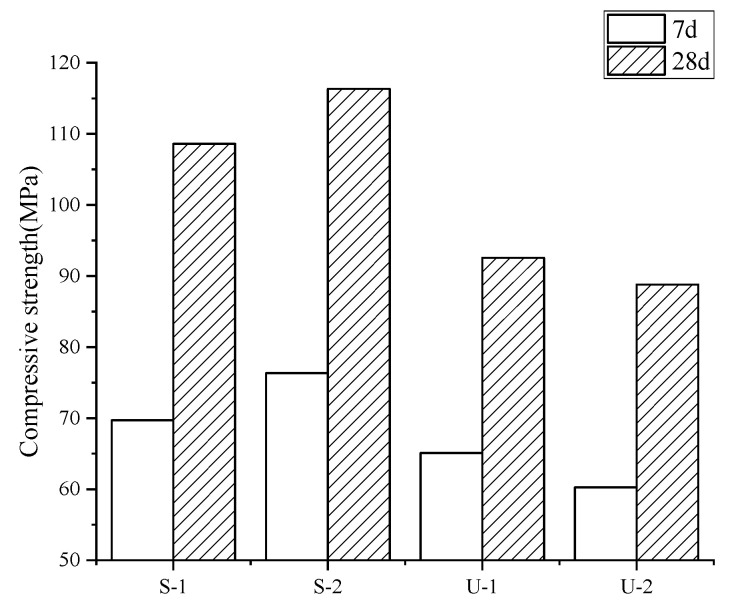
Cubic compressive strength.

**Figure 5:**
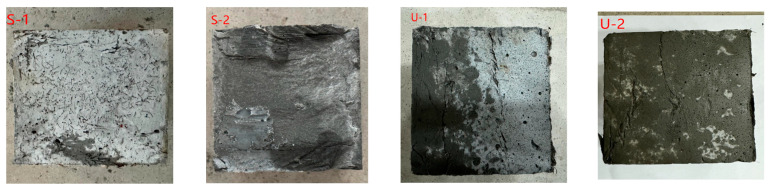
Failure mode of the cubic compressive strength test.

The test results of the cubic compressive strength are shown in Figure 4. According to Figure 4, the SIFCON specimens exhibited consistently higher compressive strength compared to the UHPC specimens. At 7 days of curing, all specimens achieved compressive strengths exceeding 70 MPa, and the strength disparity between the two increased with prolonged curing. For UHPC specimens, although the same volume of steel fibers is added, after adding RSFs (Group U-1), the cubic compressive strength is slightly higher than that of the group with ISFs added (Group U-2); it increased from 60.2 MPa to 65.1 MPa, and there is no significant change with the increasing curing age. The underlying reason is that recycled steel fibers (RSFs), characterized by their non-uniform lengths, irregular geometries, and curved configurations, develop a significantly stronger mechanical interlocking effect through “fiber entanglement” compared to the relatively weaker interfacial interlock provided by straight industrial steel fibers (ISFs) without hooked ends. This enhanced interlocking mechanism makes the RSFs more resistant to pulling out from the cementitious matrix [18]. For SIFCON specimens, with the increasing fiber dosage, the cubic compressive strength will increase significantly. The cubic strength growth rate reaches 6.7% at the age of 7 days of curing and 12.5% at the age of 28 days of curing, indicating that the steel fibers make a significant contribution to the cubic compressive strength. Figure 5 shows the failure mode of the cubic compressive strength test.

Figure 5 shows the failure mode of the cubic compressive strength test. From Figure 5, two distinct types of splitting failure modes can be observed. For the UHPC specimens, as the loading process advanced, the failure process exhibited relatively greater ductility. Crack propagation was effectively constrained, allowing the specimens to retain substantial structural integrity throughout the loading phase. Ultimately, through cracks formed, yet the specimens still exhibited significant ductile behavior, highlighting their unique capacity to sustain deformation before complete failure. For the SIFCON specimens, during loading, fine surface cracks first emerged, followed by surface segment spalling. Even post-failure, the SIFCON specimens retained residual load-bearing capacity, demonstrating that steel fibers significantly enhanced the toughness.

### 3.2. Uniaxial Compression Test Results

**Figure 6:**
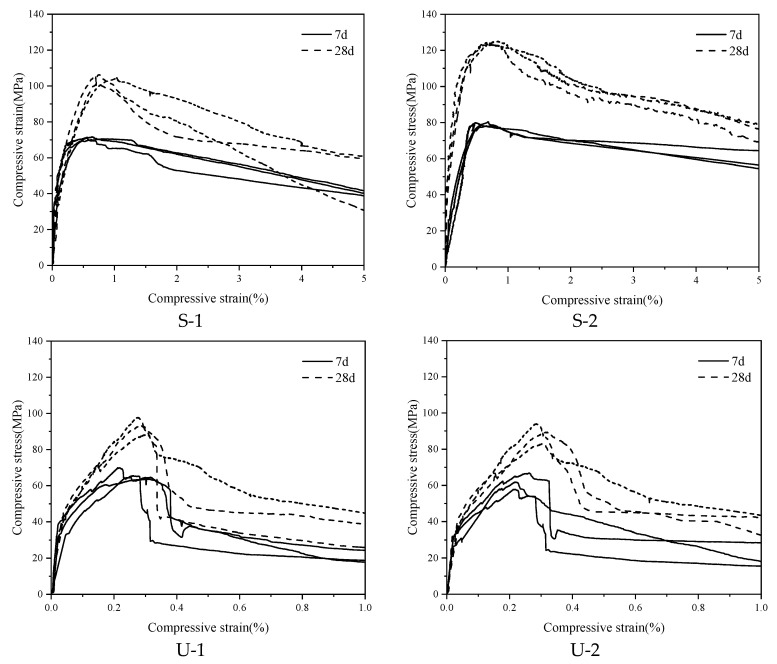
Compressive stress–strain curve of cylindrical specimens.

Uniaxial compression tests were carried out on different specimens after 7-day and 28-day curing, and the corresponding compressive stress–strain curves are shown in Figure 6.

**Figure 7:**
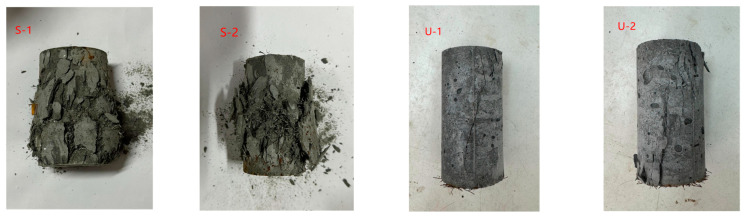
Failure mode of the cylindrical compressive test.

As shown in Figure 6, at the curing age of 28 days, the average peak compressive strengths of the S-1 and S-2 specimens reached 108.4 MPa and 126.7 MPa, respectively, significantly exceeding those of the U-1 and U-2 specimens, which were 91.3 MPa and 89.9 MPa. These results demonstrate the superior compressive performance of SIFCON compared to UHPC. Furthermore, with increasing strain, the SIFCON specimens did not exhibit abrupt failure; instead, they maintained a certain level of load-carrying capacity post-peak, indicating excellent deformation compatibility and energy dissipation capacity. The high residual strength observed is one of the distinguishing mechanical advantages of SIFCON [11]. In contrast, UHPC specimens showed a typical brittle failure mode after reaching peak stress, characterized by a steep post-peak decline and the absence of noticeable plastic deformation, reflecting the limited ductility of the material. Figure 7 shows the failure mode of the cylindrical compressive test.

Under the same fiber dosage, the increase in the compressive strength of UHPC by ISFs is not significant compared with that by RSFs. The difference is only 2.2% on average at the curing age of 28 days. This is mainly because, among the recycled tire steel fibers with the same volume of fiber dosage, there are some short fibers with a length of less than 3 mm, which makes the fibers unable to fully play the bridging role in the matrix.

After reaching the peak compressive stress, Group S-1 has a gentle decline, showing higher toughness and better ductile failure characteristics. However, Group S-2 has an obvious decline, and there are fluctuations during the decline process, indicating that the material can still maintain a certain residual bearing capacity after the peak stress, but the plastic deformation ability of the descending section is limited. The peak compressive stress of Group S-2 is slightly greater than that of Group S-1, and the effect of fiber dosage on improving the compressive strength is not obvious. This shows that under a relatively high fiber dosage, increasing the fiber dosage of SIFCON has a small effect on improving the compressive strength.

Figure 7 shows the failure mode of the cylindrical compressive test. From Figure 7, it can be seen that the failure modes of the cylindrical compressive test for UHPC specimens and SIFCON specimens differ significantly. For UHPC specimens, fine cracks initiated first with loading progression, followed by rapid crack propagation. The cracks vertically traversed the entire specimen, leading to a highly sudden failure process. For SIFCON specimens, as loading advanced, numerous micro-cracks first developed on the specimen surface, accompanied by spalling of surface segments. Crack propagation proceeded at a slower pace, with fractured blocks maintaining structural integrity via extensive fiber bridging. Moreover, specimens retained residual load-bearing capacity after failure.

### 3.3. Uniaxial Tensile Test Results

**Figure 8:**
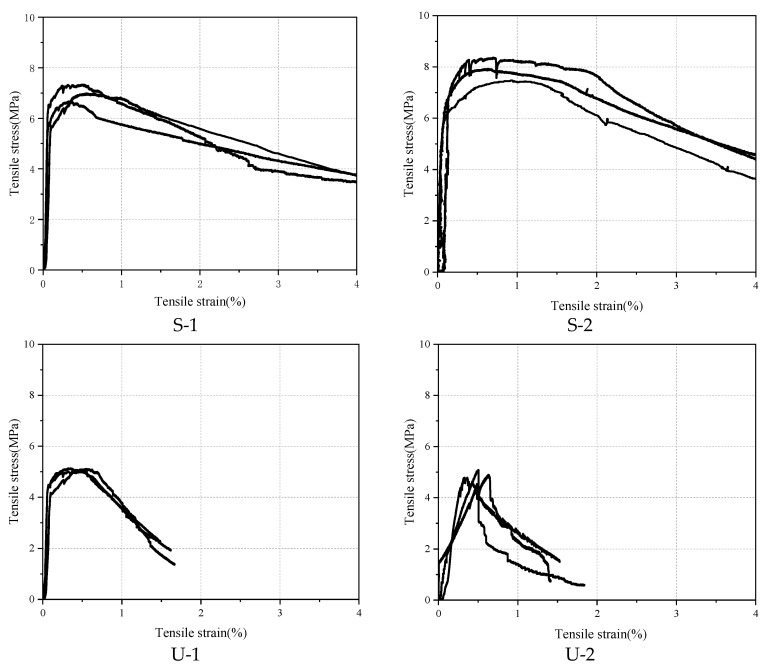
Tensile stress–strain curve.

Uniaxial tensile tests were carried out after 28-day curing, and the corresponding tensile stress–strain curves are shown in Figure 8.

As seen from Figure 8, the ultimate tensile stresses of Groups S-1 and S-2 are relatively high, with the average ultimate tensile stresses being 6.76 MPa and 8.24 MPa, respectively, which are much higher than those of Groups U-1 and U-2. It can be seen that by using the infiltration casting method, the fibers are evenly arranged and not easy to agglomerate, which can greatly increase the fiber dosage. Therefore, the fiber network in the concrete is denser, forming a strong fiber–matrix interfacial bonding effect, which can better bear and disperse the stress, inhibit the development of cracks, and thus improve the tensile strength. Groups S-1 and S-2 can maintain a relatively high tensile strength under high-strain conditions. When the tensile strain reaches 4%, the tensile strength can still reach about 4 MPa, and the specimens do not completely break, indicating that they have excellent toughness.

Under the same fiber dosage, the ultimate tensile stresses of group U-1 (4.98 MPa) and group U-2 (5.21 MPa) differ by only 0.23 MPa, indicating a negligible variation. This suggests that recycled steel fibers (RSFs) provide a tensile strength contribution comparable to that of industrial steel fibers (ISFs). The slight difference between the two groups implies that the geometrical and mechanical characteristics of RSFs are sufficient to ensure effective stress transfer and crack-bridging capacity under tensile loading [19]. This finding is particularly significant, as RSFs—typically featuring more irregular shapes and higher surface roughness due to the mechanical shredding process—may enhance mechanical interlock with the cementitious matrix. Furthermore, the experimental results reinforce the feasibility of employing RSFs as a sustainable alternative to conventional fibers in cementitious composites.

Post-crack energy refers to the energy absorbed by a material from the initiation of a crack to its complete fracture. It is a key indicator for measuring the fracture toughness and ductility of materials. For uniaxial tensile tests, the area under the stress–strain curve from the crack initiation point to the fracture point (i.e., energy density) is utilized to calculate Gpost, as shown in Equation (1),

(1) $$G_{post}=\int_{\varepsilon_{cr}}^{\varepsilon_{\max}}\sigma (\varepsilon )\;d\varepsilon$$

where *ε*_max_ is the fracture strain, *ε*_cr_ is the crack initiation strain, and *σ*(*ε*) is the stress–strain function.

**Figure 9:**
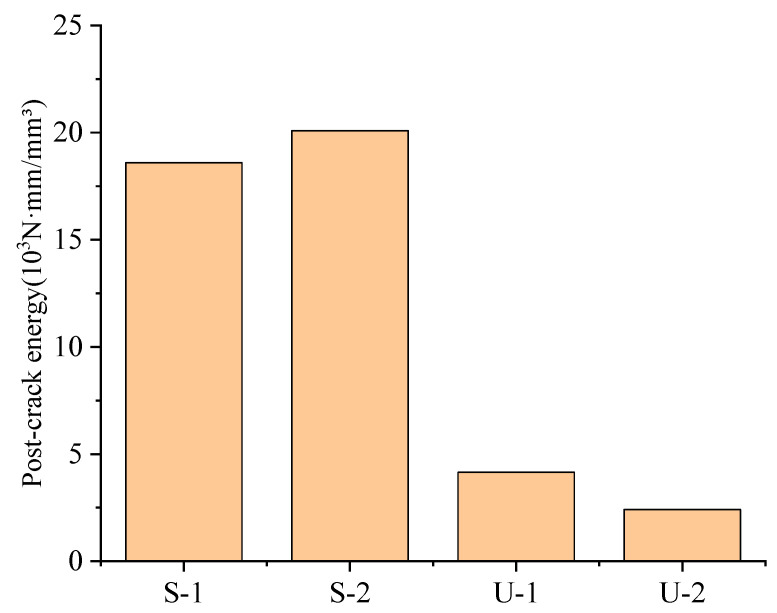
Post-crack energy.

According to Equation (1), the corresponding post-cracking energy of four types of specimens can be calculated, as shown in Figure 9.

It can be clearly observed that the energy absorption capacity of SIFCON groups during the crack propagation stage is significantly superior to that of UHPC. Specific data show that under the conditions of high fiber volume fraction and uniform distribution, SIFCON exhibits more excellent post-crack toughness. As the fiber content increases from 7% to 10% (i.e., from group S-1 to group S-2), the post-crack energy of SIFCON increases from 18.59 × 10^3^ N·mm/mm^3^ to 20.09 × 10^3^ N·mm/mm^3^, but the increase is relatively limited. This phenomenon indicates that when the fiber content is at a high level, the strengthening effect of continuing to increase the fiber dosage gradually tends to saturate.

In contrast, the post-crack energy of group U-2 (using RSFs) in UHPC materials shows a significant downward trend compared with group U-1 (using ISFs). This result reveals that replacing ISFs with RSFs in the UHPC system will lead to a significant reduction in the post-crack toughness. The main reasons are that the RSFs have irregular geometric shapes, large dimensional discreteness, and poor bonding performance with the matrix, thereby weakening the strengthening effect of the fibers on the matrix.

**Figure 10:**
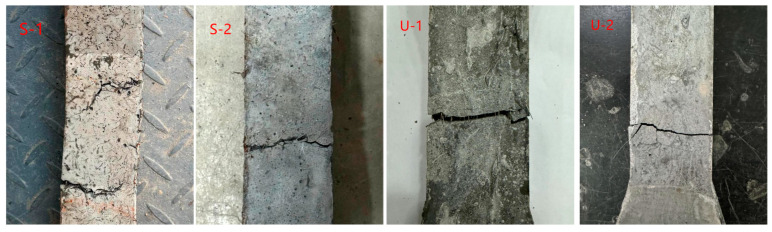
Failure mode of the tensile strength test.

Figure 10 shows the failure mode of the tensile strength test. It can be observed that the specimens mainly failed due to axial cracking along the loading direction. With increasing strain, fine cracks are first initiated in the mid-region or weak zones of UHPC specimens. These cracks then propagated rapidly and penetrated transversely, culminating in fracture failure characterized by a smooth fracture surface and a highly abrupt failure process. In contrast, SIFCON specimens exhibited more pronounced ductile failure characteristics. Crack propagation was accompanied by behaviors such as fiber bridging, pull-out, and fracture. The fracture surface was rough, and specimens retained partial integrity post-failure. The slower crack propagation rate underscored the significant enhancement of tensile properties.

## 4. Carbon Emission Assessment

The Life Cycle Assessment (LCA) method, as a systematic environmental impact assessment tool, has been widely applied to evaluate the overall environmental footprint of building materials and structures [20,21,22]. In this paper, the LCA method is adopted to evaluate the impact of steel fibers on the carbon emissions of high-performance cement-based materials. When calculating, the following two functional units are considered, namely 1 m^3^ of material and a comprehensive analysis of the influence of mechanical properties and material volume. It mainly takes into account three stages: raw material production, raw material transportation, and cement-based material preparation. When calculating carbon emissions, CO_2_ is taken as the benchmark, and different greenhouse gases are converted into CO_2_ equivalents according to their global warming potentials over a certain period of time. The carbon emission calculation formula of the cement-based material is as follows:

(2) $$C=C_{\mathrm{rm}}+C_{t}+C_{p}$$

(3) $$C_{\mathrm{rm}}=\sum_{i}Q_{i}\times RM_{C-i}$$

(4) $$C_{t}=\sum_{i}Q_{i}\times D_{i}^{j}\times TC_{i}^{j}$$

where $C_{\mathrm{rm}},C_{t},C_{p}$ represents the carbon emissions in the raw material production, transportation, and preparation stages, respectively. *C* is the total carbon emissions in the production stage of high-performance cement-based materials. $Q_{i}$ represents the usage amount of the *i-th* material, $RM_{C-i}$ represents the carbon emission per unit mass of the *i-th* material. $D_{i}^{j}$ represents the distance of transporting the *i-th* material to the project site by using the transportation equipment *j*. $TC_{i}^{j}$ represents the carbon emissions generated when transporting 1 ton of the *i-th* material by the transportation equipment *j* travels 1 km.

The carbon emissions in the raw material production stage can be determined according to the mix proportion of various types of high-performance cement-based material and the actual consumption quantity of raw materials and by multiplying with the determined corresponding carbon emission coefficient. The carbon emissions in the transportation stage can be determined according to the weight of various raw materials, the distance of transporting various raw materials to the project site, and the transportation equipment used.

### 4.1. Life Cycle Inventory

The carbon emission factors of the raw materials involved in this paper mainly come from the China Life Cycle Database (CLCD) [23] and the Ecoivent database [24], as shown in Table 7.

It is worth noting that RSFs are typical waste. Their effective recycling and utilization not only help reduce the amount of waste landfilled and alleviate the pressure on the increasingly scarce land resources but also avoid the permanent occupation of land and potential environmental pollution problems caused by long-term landfilling. Incorporating RSFs into concrete as reinforcement materials realizes the recycling of waste, avoids landfilling, and reduces carbon emissions. According to the research of Zhang et al. [25], the carbon emission factor during the landfilling process of RSFs is taken as 0.01417 kg CO_2_/kg. Therefore, when calculating the carbon emission, this carbon emission reduction effect should be taken into consideration.

At present, the main short-distance transport tools in China are trucks or freight vehicles. According to CLCD, the carbon emission factor of diesel trucks/freight vehicles is 0.16 kg (CO_2_)/(km·t). In addition, the transport distance of raw materials will affect the carbon emission calculation result. Note that the transport distance is closely related to the actual situation, which can only be determined after investigation. According to the literature and on-site investigations, the transport distances of various raw materials involved in this paper are shown in Table 8. The high-performance cement-based materials preparation stage mainly includes three processes, mixing, pouring, and vibrating, with the corresponding carbon emission factors being 0.7 kg/m^3^, 0.2 kg/m^3^, and 2.9 kg/m^3^, respectively. It should be noted that during the preparation process of SIFCON, there is no need for mixing steel fibers and vibrating.

### 4.2. Volumetric Carbon Emission

**Figure 11:**
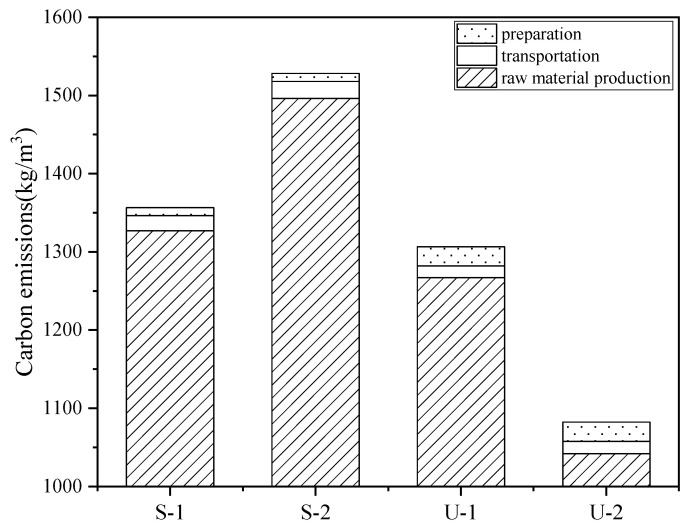
Volumetric carbon emissions.

Based on the mix proportion data and the corresponding carbon emission factors, the volumetric carbon emissions can be calculated, as shown in Figure 11.

**Figure 12:**
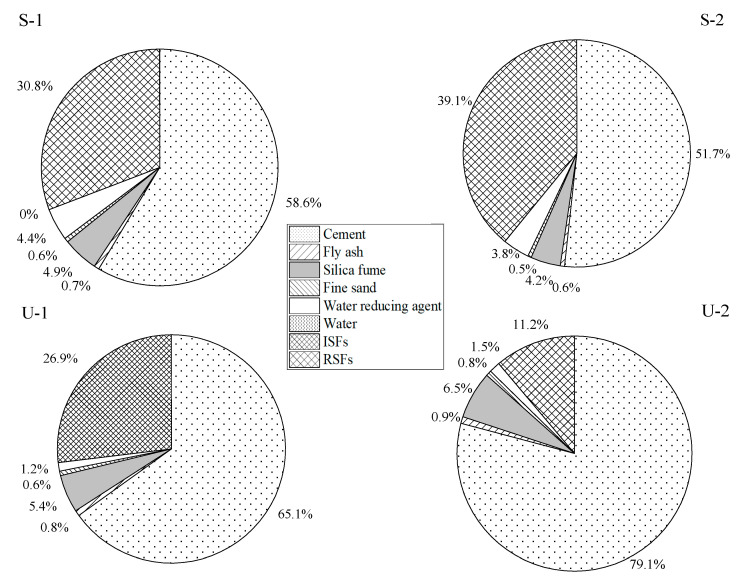
Carbon emissions from the raw material production stage.

It can be seen from Figure 11 that the volumetric carbon emissions of S-1 and S-2 both exceed 1300 kg/m^3^, while the carbon emissions of U-1 and U-2 are approximately 1200 kg/m^3^ and 1100 kg/m^3^, respectively. The main reason is that the SIFCON group uses a large amount of fibers, which additionally generates corresponding carbon emissions. In the UHPC group, since the carbon emission factor of RSF is only one-third of that of ISF, the volumetric carbon emissions of U-2 are obviously lower than those of U-1. The raw material production stage is the main source of carbon emissions, accounting for more than 95% of the total emissions, and the transport stage and the preparation stage have less impact on the total carbon emissions. To analyze the carbon emission contributions of various raw materials in detail, the carbon emissions in the raw material production stage were calculated, and the results are shown in Figure 12.

From Figure 12, it can be seen that for SIFCON and UHPC, cement production accounts for the main contribution of carbon emissions during the raw materials production stage, which is 58.6%, 51.7%, 65.1%, and 79.1% of the four groups, respectively. In addition, the amount of steel fibers used in SIFCON is more than three times that in UHPC, but the resulting carbon emissions do not increase exponentially. This indicates that although a large amount of steel fibers are used in SIFCON, it will not lead to a significant increase in carbon emissions.

### 4.3. Analysis of Carbon Emissions per Unit Strength

**Figure 13:**
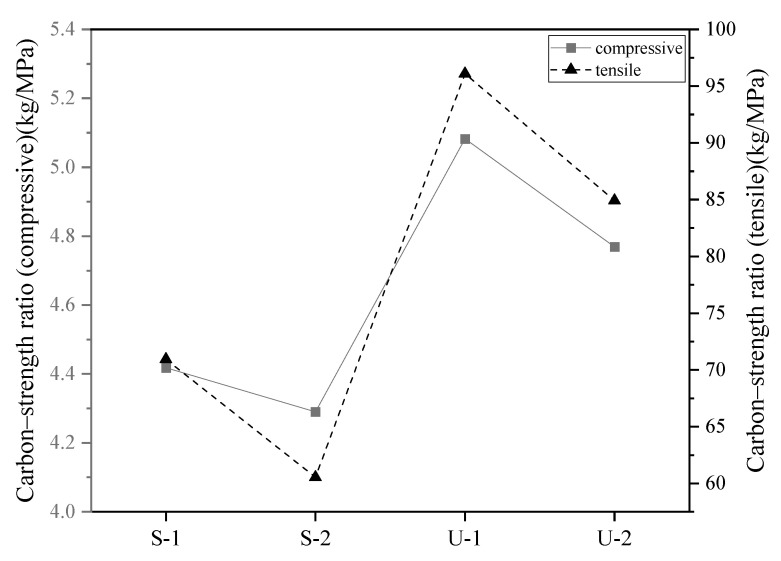
Carbon/strength ratio.

The carbon/strength ratio can be defined as the carbon emissions per unit strength. A lower carbon/strength ratio indicates that the cement-based material has a relatively small environmental impact while ensuring its strength, making it a more environmentally friendly option. Figure 13 calculates the corresponding carbon/strength ratios based on the compressive strength and tensile strength, respectively.

As can be seen from Figure 12, whether calculated based on the tensile strength or the compressive strength, the carbon/strength ratio of the SIFCON group is significantly lower than that of the UHPC group. Regarding the compressive carbon/strength ratio, the values are 4.418 kg/MPa (S-1) and 4.290 kg/MPa (S-2), respectively, indicating that SIFCON has the characteristics of lower carbon emissions while ensuring high compressive strength and has a relatively small impact on the environment. In contrast, the compressive carbon/strength ratios of the UHPC group are 5.082 kg/MPa (U-1) and 4.769 kg/MPa (U-2), respectively, reflecting that the UHPC material has a higher carbon dioxide emission per unit of strength and relatively weaker comprehensive sustainability. Similarly, the tensile carbon/strength ratio also shows the same trend of change.

The tensile carbon/strength ratios of the SIFCON group are all below 70 kg/MPa. Notably, the S-2 mixture, incorporating a higher volume fraction of RSFs compared to S-1, exhibits a further reduction to 60.54 kg/MPa, representing a 14.28% decrease. This suggests that increasing the content of RSF can effectively enhance both tensile performance and carbon efficiency. In contrast, the UHPC groups show tensile carbon/strength ratios exceeding 85 kg/MPa, indicating inferior environmental performance. The U-1 mixture, which employed ISFs, exhibited the highest tensile carbon/strength ratio among all groups. In comparison, U-2 achieved a certain degree of carbon emission reduction while maintaining relatively high tensile strength, highlighting the environmental advantages and sustainability potential of RSFs.

It is worth noting that the reported carbon emission value of 1300 kg/m^3^ for S-1 and S-2 is higher than the typical range documented in references, for example, Wang et al. [13] reported that UHPC incorporating 2% RTSF with compressive strength of 83 MPa and tensile strength of 2.7 MPa, exhibited a carbon dioxide emission of 929.64 kg CO_2_/m^3^, with a carbon/strength ratio (compressive) of 11.2 kg/MPa and a carbon/strength ratio (tensile) of 344.3 kg/MPa. The main reason arises from the specific characteristics of SIFCON material investigated in this study, which has a significantly higher content of cement and binder. In addition, its fiber content is high as well. These higher material contents contribute to high carbon emissions.

Although the carbon dioxide emission of S-1 and S-2 is higher than that of Wang et al.’s result, S-1 and S-2 have significantly superior mechanical properties. When taking the mechanical properties into consideration, the carbon/strength ratios of S-1 and S-2 are far less than that of Wang et al.’s result. In our work, the carbon/strength ratio (compressive) of S-1 and S-2 is 4.418 kg CO_2_/MPa and 4.290 kg CO_2_/MPa, respectively. In addition, the carbon/strength ratios (tensile) of S-1 and S-2 are both below 70 kg/MPa, which is about 1/5 of the carbon/strength ratio (tensile) reported by Wang et al. [13].

## 5. Conclusions and Future Perspectives

### 5.1. Conclusions

By investigating the effects of steel fiber content, fiber type, and casting method on the mechanical properties and carbon emissions of concrete, the following conclusions are drawn:

(1) The compressive strength of SIFCON increases markedly with higher RSF content, exceeding that of conventional UHPC by over 35%, while its tensile strength is about 60% higher. SIFCON also shows enhanced ductility and energy absorption, evidenced by higher post-peak residual strength and a slower stress drop.

(2) The tensile strength improvement in concretes with RSFs and ISFs is comparable, indicating that RSFs can replace ISFs without compromising tensile performance. This supports the feasibility of using RSFs as a cost-effective and sustainable alternative in fiber-reinforced concrete

(3) LCA results show that RSFs significantly reduce carbon emissions, with a 65.9% lower unit carbon footprint compared to ISFs. Although SIFCON emits more total CO_2_ emissions than UHPC due to higher fiber content, its mechanical performance gain results in a notably lower carbon intensity, demonstrating its efficiency in emission reduction.

### 5.2. Future Perspectives

The utilization of RSFs in producing concrete through the infiltration casting technique not only showcases remarkable potential in enhancing mechanical properties but also ushers in a new era of sustainable construction. This significant enhancement in mechanical performance is attributed to the uniform dispersion of RSFs within the matrix, which effectively restrains crack propagation under load. Moreover, the environmental benefits are equally compelling. By replacing virgin steel fibers with recycled counterparts, the carbon intensity of concrete production can be reduced. The potential application prospects of RSFs in the concrete industry are extensive. Beyond road pavement, RSFs can be used in bridge decks, building facades, and precast concrete elements, where their enhanced durability and crack resistance can significantly extend the service life of structures. This not only reduces the frequency of repairs and replacements but also minimizes the overall demand for construction materials, thereby further reducing the environmental footprint.

In the context of global efforts to build waste-free cities, the adoption of RSFs aligns perfectly with the principles of circular economy. With continuous advancements in material science and manufacturing technologies, RSFs are expected to play an increasingly pivotal role in promoting the development of waste-free cities, driving the construction industry towards a more environmentally friendly and resource-efficient future. To further validate the enhancement effects of RSFs, future research should include control mix tests without fibers, which would allow for more precise quantification of their contribution to the mechanical properties of concrete.

## Figures and Tables

**Table 1:** Basic properties of cement.

Loss on Ignition/%	Sulfur Trioxide/%	Magnesium Oxide/%	Chloride ion Content/%	Standard Consistency/%	Specific Surface Area/(m^2^/kg)
1.98	2.21	1.98	0.013	28.2	397

**Table 2:** Chemical composition and physical properties of silica fume.

Chemical Composition	Loss on Ignition/%	Moisture Content/%	Residue on 45 μm Sieve/%
SiO_2_ ≥ 87.2 K_2_O ≤ 0.86 Na_2_O ≤ 0.13	≤3.63	0.77	1.61

**Table 3:** Chemical composition of Class I fly ash (%).

SiO_2_	Al_2_O_3_	MgO	CaO	Fe_2_O_3_	K_2_O	SO_3_
47.66	20.81	1.51	11.51	9.84	1.65	0

**Table 4:** Properties of water reducing agent.

Appearance	Loss on Drying/%	Bulk Density/(kg/m^3^)	pH Value
yellow powder	≤2	300–600	6.5–8.5

**Table 5:** Physical properties of steel fibers.

Type	Morphology	Length/mm	Diameter/mm	Elastic Modulus/GPa	Tensile Strength/MPa	Density/(kg/m^3^)
RSF	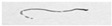	3~18	0.2	200	2000	7800
ISF	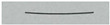	13	0.2	200	2000	7800

**Table 6:** Mix proportion/(kg/m^3^).

Group	Cement	FA	SF	Sand	WRA	Water	ISF	RSF	Fiber Volume Fraction
S-1	915.0	185.6	185.6	335.0	19.0	642.4	0.0	545.0	7%
S-2	910.3	181.6	181.6	330.0	18.5	638.4	0.0	780.0	10%
U-1	970.1	194.3	194.3	354.7	5.0	680.0	155.0	0.0	2%
U-2	970.1	194.3	194.3	354.7	5.0	680.0	0.0	155.0	2%

**Table 7:** Carbon emission factors of the raw materials (kg CO_2_/kg).

Material	Inventory Source	Carbon Emission Factor	Material	Inventory Source	Carbon Emission Factor
cement	CLCD	0.85	water reducing agent	Ecoinvent	3.09
fly ash	CLCD	0.05	water	Ecoivent	0.000329
silica fume	CLCD	0.35	ISF	Ecoinvent	2.2
fine sand	Ecoivent	0.01172	RSF	Ecoinvent	0.75

**Table 8:** Transport distance of raw materials (km).

Raw Materials	Cement	Fly Ash	Silica Fume	Fine Sand	Water Reducing Agent	ISF	RSF
Transport distance (km)	30	60	60	50	60	50	70

## Data Availability

The original contributions presented in this study are included in the article. Further inquiries can be directed to the corresponding author.
